# Diversity and Origin of Quill Mites of the Subfamily Syringophilinae (Acariformes: Syringophilidae) Parasitising the True Finches (Passeriformes: Fringillidae)

**DOI:** 10.3390/ani15213227

**Published:** 2025-11-06

**Authors:** Maciej Skoracki, Markus Unsoeld, Roland R. Melzer, Stefan Friedrich, Bozena Sikora

**Affiliations:** 1 Department of Animal Morphology, Faculty of Biology, Adam Mickiewicz University, 61-614 Poznań, Poland; 2 Ornithology Section, Bavarian State Collection for Zoology, Bavarian State Collections of Natural History (SNSB), 81247 Munich, Germany; unsoeld@snsb.de; 3 Arthropoda varia Section, Bavarian State Collection for Zoology, Bavarian State Collections of Natural History (SNSB), 81247 Munich, Germany; melzer@snsb.de (R.R.M.); friedrich@snsb.de (S.F.); 4 Faculty of Biology, Ludwig Maximilian University of Munich, 82152 Planegg-Martinsried, Germany; 5 GeoBio-Center, Ludwig Maximilian University of Munich, 80333 Munich, Germany

**Keywords:** Acari, Aves, biodiversity, birds, ectoparasites, Fringillidae, Syringophilidae

## Abstract

Quill mites belonging to the family Syringophilidae are highly specialised external parasites that live inside feather quills. However, parasitologists have limited knowledge about their diversity and patterns of host specificity, particularly regarding how restricted syringophilids are in their choice of avian hosts across different bird lineages. This research presents the first complete overview of mites of the subfamily Syringophiline found on True Finches (Fringillidae). Based on a combination of all published records and extensive examinations of museum specimens of fringillids housed at the Bavarian State Collection of Zoology in Munich, Germany, we report 20 mite species from 5 genera parasitising 51 finch species across all 3 subfamilies: Fringillinae, Euphoninae, and Carduelinae. Four species are described here as new to science: *Aulobia ruppelae* sp. n., *Aulonastus ritzerfeldi* sp. n., *Torotrogla enucleator* sp. n., and *T. janhafti* sp. n. Herein, we also recognised that most syringophilid mites associated with the True Finches, parasitise hosts belonging to the same or phylogenetically closely related genera, suggesting strong phylogenetic conservatism. The variation in host range and speciation rate across mite genera suggests that quill mite diversification does not always proceed synchronously with host evolution. These discoveries offer an important understanding of how host–parasite relationships evolve over time.

## Simple Summary

Finches are a large and diverse group of birds found worldwide, yet little is known about the parasites of the family Syringophilidae that live inside their quill feathers. These parasites form close and often long-lasting relationships with their bird hosts. In this study, we investigated which species of syringophilid mites inhabit finches and how specific these mites are to particular bird species. We identified 20 different mite species on 51 finch species, including 4 species newly described for science. Our research indicates that most syringophilids are not strictly limited to a single bird species, but rather occupy birds that are closely related. Interestingly, we did not find any mites that switched between phylogenetically not closely related finch groups. These findings suggest that syringophilid mites and finches have evolved together over long periods of time. This kind of research helps us understand how parasites adapt to their hosts and how these hidden relationships shape biodiversity. It also shows the importance of studying old museum specimens, which can still reveal new scientific discoveries even after many decades.

## 1. Introduction

Mites associated with birds comprise representatives of numerous families and display a remarkable diversity of ecological strategies, ranging from commensalism, in which the mite benefits without causing measurable harm to its host, to parasitism, which can lead to direct damage through feeding on host tissues or resources [1]. Over 2500 mite species from about 40 families are closely associated with birds, occupying virtually every conceivable niche on the host body, including the skin, respiratory passages, and feathers [2]. Among the most intriguing groups are the quill mites of the family Syringophilidae (Prostigmata: Cheyletoidea). As their name suggests, these mites inhabit the interior of feather quills (calamus), a microhabitat that also harbours members of several, often distantly related, mite families, such as the astigmatan families Ascouracaridae, Syringobiidae, and Kiwilichiidae belonging to the superfamily Pterolichoidea [3,4,5,6]; Apionacaridae, Dermoglyphidae, Gaudoglyphidae, and Laminosioptidae belonging to the superfamily Analgoidea [7,8,9,10,11,12]; and members of the tribes Cheletosomatini and Metacheyletiini of the prostigmatan family Cheyletidae, which are close relatives of the family Syringophilidae [13,14,15,16].

Unlike other quill mites, syringophilids are unique in feeding on the fluid tissues of their avian hosts, piercing the calamus wall with elongated, needle-like chelicerae to access host nutrients, lymph, or blood [17]. This specialised feeding mode, combined with pronounced host and habitat specificity, has driven their diversification into one of the most taxonomically rich groups of quill-inhabiting mites, with approximately 400 described species in 63 genera and 2 subfamilies, recorded from hosts representing 27 avian orders [18,19]. However, historical sampling of this mite fauna has often been fragmentary, with new species described opportunistically and without a broader taxonomic or ecological framework. Only recently have systematic surveys begun to target entire avian genera [20,21], families [22,23] or orders [24,25], revealing substantial hidden diversity and clarifying host–parasite relationships. As part of the continuing effort to characterise syringophilid diversity in passerines, the most species-rich avian order, this study concentrates on their occurrence on the representatives of the family Fringillidae (Passeriformes). This group of birds represents a widespread lineage of songbirds, making it an excellent model for exploring patterns of host specificity, diversity, and distribution of syringophilid mites.

The True Finches (Fringillidae) represent one of the most morphologically and ecologically diverse families among oscine passerines. Despite wide variation in plumage and bill shape, they share several core traits, including a predominantly granivorous and frugivorous diet that extends even to feeding nestlings. Their broad ecological tolerance allows them to inhabit environments ranging from arid scrublands to alpine zones and tropical rainforests [26]. The Fringillidae family is nested within the superfamily Passeroidea, one of the most extensive and evolutionarily successful radiations among oscine passerines. Although early molecular data suggested a sister-group relationship with Passeridae [27], most subsequent analyses support a closer affinity between Fringillidae and the New World nine-primaried oscines (Passerellidae, Thraupidae, Icteridae, Cardinalidae, and Parulidae) [28,29,30,31,32,33]. Within Fringillidae, three subfamilies are currently recognised: Fringillinae represents the most basal lineage; Euphoniinae, formerly classified within Thraupidae; and Carduelinae, the most species-rich group [26,33,34].

The wide distribution and complex evolutionary history of Fringillidae, most diverse in the Old World, particularly in Eurasia and Africa, make its members especially valuable for studying host–parasite associations. Among their diverse ectoparasites, quill mites represent a group whose diversity and host relationships remain incompletely understood. To address this gap, we present a comprehensive assessment of Syringophilinae, one of the two subfamilies of syringophilid mites associated with the True Finches, focusing on species richness, patterns of host specificity, and implications for host–parasite coevolution. By examining a broad taxonomic spectrum of Fringillidae, we provide new data that advance an integrated understanding of quill mite diversity and the evolutionary dynamics of finch–mite interactions. In addition to ecological and evolutionary analyses, we also provide an identification key to the syringophilid species parasitising Fringillidae, updated diagnoses of previously known species, and descriptions of four new species. These systematic contributions form the basis for a more accurate taxonomic and evolutionary framework of these mites and their hosts.

## 2. Materials and Methods

The newly acquired material of quill mites was obtained from dry-preserved bird specimens deposited at the Bavarian State Collection of Zoology (SNSB-ZSM) in Munich, Germany. From each finch specimen, a standardised set of feathers was selected for examination, i.e., approximately ten contour feathers from the cloaca region, along with two upper-tail coverts, two under-tail coverts, and one secondary wing covert. Selected feathers were screened for the presence of syringophilid mites. The infested quills were placed in Nesbitt’s solution and kept at room temperature for three days to allow softening of mite tissues [35,36]. Subsequently, each quill was longitudinally dissected with fine-tipped forceps to extract the mites. The recovered specimens were rinsed in 70% ethanol and mounted on permanent microscope slides in Hoyer’s medium [37] for morphological analysis. Slide-mounted mites were examined using a ZEISS Axioscope light microscope (Carl Zeiss AG, Oberkochen, Germany) fitted with differential interference contrast (DIC) optics and a camera lucida.

In the description, the terminology for idiosomal setation adheres to Grandjean’s system [38], as modified for Prostigmata by Kethley [39]. Leg chaetotaxy follows Grandjean’s nomenclature [40], while general morphological terminology is based on Kethley [41] and Skoracki [42]. All measurements are expressed in micrometres, with ranges for paratypes provided in brackets following the holotype data.

Collected mite specimens have been deposited in two institutional collections: the Department of Animal Morphology at Adam Mickiewicz University in Poznań (AMU), Poland, and the Bavarian State Collection for Zoology (SNSB-ZSM), Germany.

Avian taxonomy and nomenclature adhere to the classifications of Winkler et al. [26] and the Clements et al. [43].

The geographical distributions of host species were determined using BirdLife International data [44,45]. The delineation of zoogeographic regions relies on the frameworks proposed by Holt et al. [46] and Ficetola et al. [47]

Host specificity for particular mite species follows Caira et al. [48] and Skoracki et al. [49]. In this context, we delineate four distinct categories: monoxenous parasites which are limited to a singular host species; oligoxenous parasites inhabiting multiple host species, yet all are contained within the same genus; mesostenoxenous parasites are linked with host species from various genera, while still being classified within the same subfamily; and metastenoxenous parasites utilise hosts from diverse subfamilies, but remain confined to the family Fringillidae.

The bar chart was generated in the R (ver. 4.0.) statistical environment [50].

## 3. Results

### 3.1. Syringophilinae Species Richness Associated with the True Finches

#### 3.1.1. *Aulobia cardueli* Skoracki, Hendricks & Spicer, 2010

Diagnosis. Female. Total body length 815–855. Infracapitulum densely punctate. Each medial branch of peritremes with 3–4 chambers, each lateral branch with 18–20 chambers. Stylophore 180 long. Propodonotal shield punctate near bases of setae *ve* and *si*. Hysteronotal shield not fused to pygidial shield, apunctate. Pygidial shield, large and punctate posteriorly. Coxal fields I–IV punctate. Fan-like tarsal setae III–IV *p*′ and *p*″ with 7–8 tines. Lengths of setae: *vi* 30–40, *ve* 40–60, *si* 50–75, *se* 205–225, *c1* 215–245, *c2* 210–235, *d1* 160–190, *d2* 160–185, *e2* 140–170, *f1* 60–85, *f2* 190–210, *h1* 60–90, *h2* 295–325, *ps1* and *ps2* 25–30, *g1* and *g2* 55–60, *ag1* 70–75, *ag2* 75–80, *ag3* 100–115, *3b* 35–40, *3c* 70–80.

##### Hosts and Distribution

Mesostenoxenous parasite recorded from representatives of the subfamily Carduelinae in the Holarctic region: Redpoll *Acanthis flammea* (Linnaeus) in Poland, Slovakia and Germany [42,51]; European Goldfinch *Carduelis carduelis* (Linnaeus) in Kazakhstan and Poland [42,52]; Citril Finch *Carduelis citrinella* (Pallas) in Poland [42]; Twite *Linaria flavirostris* (Linnaeus) in Poland and Germany [42,51]; Lesser Goldfinch *Spinus psaltria* (Say) in the United States [53], and Eurasian Siskin *Spinus spinus* (Linnaeus) in Russia and Kazakhstan [42].

#### 3.1.2. *Aulobia leucostictus* Skoracki, 2011

Diagnosis. Female. Total body length 700–745. Infracapitulum sparsely punctate. Each medial branch of peritremes with 3–4 chambers, each lateral branch with 11–13 chambers. Stylophore 200–215 long. Propodonotal shield punctate near bases of setae *ve* and *si*. Hysteronotal shield small and weakly sclerotised, not fused to pygidial shield, apunctate. Pygidial shield apunctate. Coxal fields I–IV punctate. Fan-like tarsal setae III–IV *p*′ and *p*″ with 9–11 tines. Lengths of setae: *vi* 45–55, *ve* 60–70, *si* 75–95, *se* 280–305, *c1* 280 315, *c2* 280–310, *d1* 210–270, *d2* 220–240, *e2* 285–205, *f1* 65–85, *f2* 200–285, *h1* 60–80, *h2* 355–395, *ps1* and *ps2* 25–35, *g1* and *g2* 65–75, *ag1* 100–115, *ag2* 80–95, *ag3* 125–135, *3b* 25, *3c* 70.

##### Host and Distribution

Monoxenous parasite associated with a member of the subfamily Carduelinae: Asian Rosy-Finch *Leucosticte arctoa* (Pallas) in Japan (current paper).

##### New Material Examined

Ex *Leucosticte arctoa* (host reg. no. SNSB-ZSM 1339, male); Japan: Hokkaido, Sapporo, May 1896, coll. A. Owston—four females and one male deposited in the AMU (reg. no. AMU-MS 24-1025-095).

##### Remarks

In a previous publication [42], the host *Leucosticte arctoa* was reported as originating from Europe. However, this is almost certainly a labelling error, as none of the currently recognised subspecies of *L. arctoa* are known to occur in the European continent. The nominate subspecies *L. a. arctoa* inhabits south-central Russian Siberia, northeastern Kazakhstan, and northwestern Mongolia; *L. a. cognata* occurs from the eastern Sayan region to central Baikal and northern Mongolia; and *L. a. sushkini* is restricted to the Khangai Mountains in west-central Mongolia [54]. Therefore, we conclude that the type host identification is valid, but the geographic label indicating “Europe” as the collection locality is incorrect. We thus exclude Europe from the distributional range of both *Aulobia leucostictus* and its host association.

#### 3.1.3. *Aulobia ruppelae* sp. n. (Figure 1 and Figure 2)

Description. Female, holotype. Total body length 870 (780–910 in 10 paratypes). Gnathostoma. Infracapitulum apunctate. Hypostomal apex without protuberances but with two pairs of unequal in size, finger-like lips. Stylophore rounded posteriorly, 240 (210–235) long, exposed portion of stylophore apunctate, 200 (180–195) long. Length of movable cheliceral digit 170 (160–170). Medial branch of peritremes with 3 chambers, each lateral branch with 10–11 delicately striated chambers. Idiosoma. Propodonotal shield well-sclerotised, 160 (155–160) long, 120 (120–125) wide on level of setae *si*, rectangular in shape, punctate between bases of setae *ve* and *si*, bearing all propodonotal setae except *c2*. Setae *se* and *c1* situated at same transverse level. Setae *vi*, *ve*, and *si* short and subequal in length. Hysteronotal shield absent. Pygidial shield with rounded anterior margin, well-sclerotised, apunctate, 100 (100–105) long and 80 (75–80) wide. Setae *f2* about 3 times longer than *f1*. Setae *f1* and *h1* subequal in length. Both pairs of pseudanal setae *ps1* and *ps2* equal in length. All coxal fields well-sclerotised and sparsely punctate. Setae *ag1* slightly (1.1–1.3 times) longer than *ag2*. Genital plate present, bearing bases of genital setae *g1* and *g2*, bases of setae *ag2* and *ag3* situated out of this plate. Both pairs of genital setae equal in length. Lengths of setae: *vi* 40 (35–45), *ve* 45 (45–55), *si* 45 (45–70), *se* (260–310), *c1* 300 (300–330), *c2* 285 (270–285), *d1* variable in length 130 (100–230), *d2* variable in length (100–210), *e2* variable in length 145 (130–215), *f1* 70 (70–90), *f2* 230 (230–250), *h1* 75 (70–90), *h2* (320–370), *ps1* and *ps2* 20 (20–30), *g1* and *g2* 60 (50–60), *ag1* 100 (105–110), *ag2* 75 (80–90), *ag3* 150 (140–150), *3b* 35 (35–40), *3c* 80 (80–95), *4b* 40 (30–40), *4c* 85 (85–90), *l’RIII* 55 (45–55), *l’RIV* 40 (30–40).

Male. Total body length 580–600 in three paratypes. Gnathostoma. Infracapitulum apunctate. Hypostomal apex without protuberances. Stylophore slightly constricted posteriorly, 190–195 long, exposed portion of stylophore apunctate, 160–165 long. Length of movable cheliceral digit 150. Medial branch of peritremes with 3–4 chambers, each lateral branch with 9–11. Idiosoma. Propodonotal shield 120–125 long, 90–95 wide on level of setae *si*, weakly sclerotised, punctate laterally, bearing bases of setae *vi*, *ve*, *si*, and *c1*, bases of setae *se* on or near this shield, bases *c2* out of this shield. Setae *se* situated anterior to level of setae *c1*. Setae *vi*, *ve*, and *si* short and subequal in length. Hysteronotal shield apunctate, weakly sclerotised, bearing bases of setae *d1* and *e2*. Hysteronotal setae *d1*, *d2*, and *e2* short. Pygidial shield small, apunctate and weakly sclerotised, anterior margin indiscernible, bearing bases of setae *f2* and *h2* on lateral margins. Aggenital series with three pairs of setae. Lengths of setae: *vi* 30–40, *ve* 30–50, *si* 20–35, *se* 165–175, *c1* 155–165, *c2* 145–160, *d1* 10–15, *d2* 20, *e2* 10–15, *f2* 20, *h2* 175, *ag1* 40–55, *ag2* 30, *ag3* 50, *3b* 25–30, *3c* 50–60, *4b* 25–30, *4c* 50–60, *l’RIII* 30–40, *l’RIV* 25–30.

##### Type Material

Female holotype and paratypes: 10 females and 3 males from Pallas’s Rosefinch *Carpodacus roseus* (Pallas) (Carduelinae) (host reg. no. SNSB-ZSM 03.1693, male); Russia: Amur Region, 19 March 1903, coll. R. Tancre.

##### Type Material Deposition

Holotype and paratypes are deposited in the SNSB-ZSM (reg. no. ZSMA20250027), except three female paratypes and one male paratype deposited in the AMU (reg. no AMU-MS 24-1025-081).

**Figure 1:**
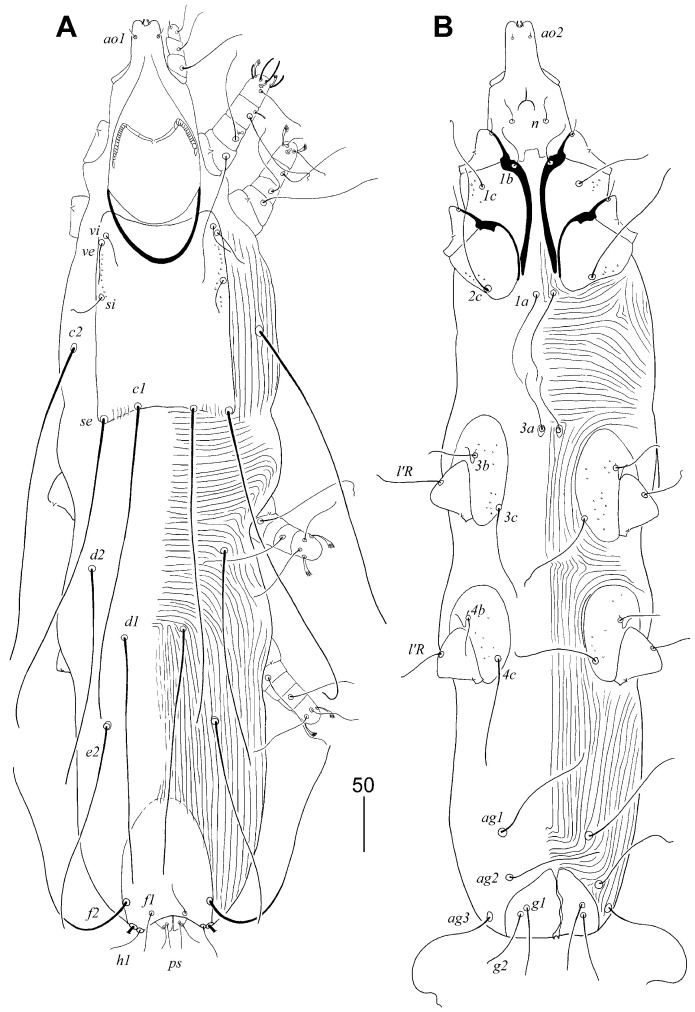
*Aulobia ruppelae* sp. n., female. (**A**) Dorsal view; (**B**) ventral view.

##### Additional Material

Ex. Beautiful Rosefinch *Carpodacus pulcherrimus* (Moore) (Carduelinae) (host reg. no. SNSB-ZSM 62.2767, male); Nepal: Khumjung, Khumbu, 20 July 1962, coll. unknown—four females and one male deposited in the AMU (reg. no. AMU-MS 24-1025-072).

##### Differential Diagnosis

*Aulobia ruppelae* sp. n. is morphologically similar to *Aulobia cardueli* Skoracki, Hendricks and Spicer, 2010. In females of both species, the hypostomal apex bears two pairs of unequal lips, and the propodonotal setae *vi*, *ve*, and *si* are short and subequal in length. This new species can be easily distinguished from *A. cardueli* by the following features: in females of *A. ruppelae* sp. n., the infracapitulum is apunctate, each lateral branch of the peritremes has 10–11 chambers, and the hysteronotal shield is absent. In contrast, in females of *A. cardueli*, the infracapitulum is densely punctate, each lateral branch of the peritremes has 18–20 chambers, and the hysteronotal shield is present.

**Figure 2:**
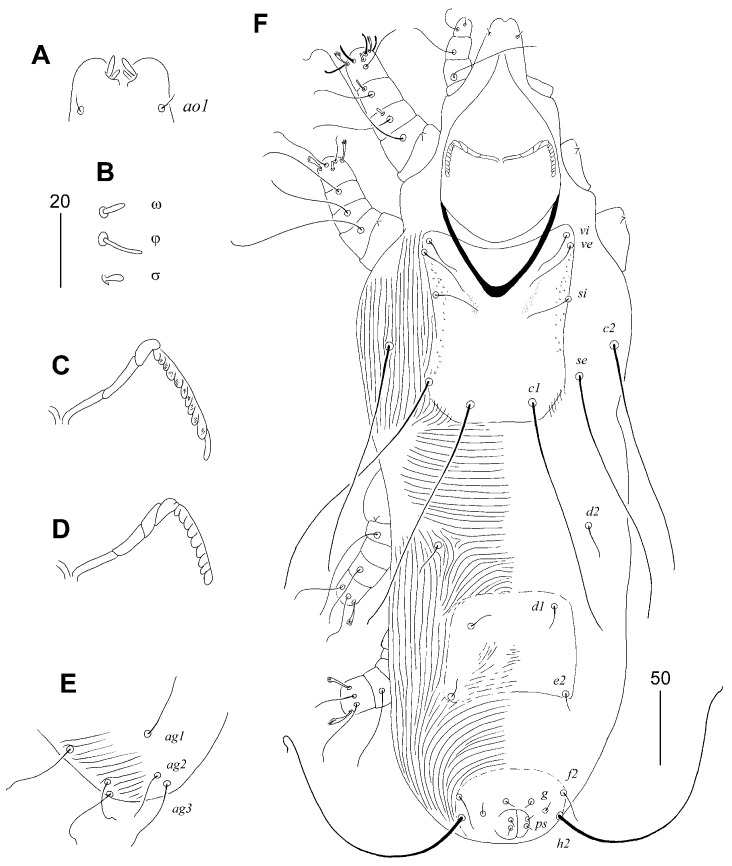
*Aulobia ruppelae* sp. n., female (**A**–**C**): (**A**) hypostomal apex; (**B**) solenidia of legs I; (**C**) peritreme. Male (**D**–**F**): (**D**) peritreme; (**E**) opisthosoma in ventral view; (**F**) body in dorsal view. Scale bars: (**A**–**D**) = 20 µm, (**E**,**F**) = 50 µm.

##### Etymology

This species is named in honour of Barbara Ruppel, a scientific illustrator with a deep interest in nature. She has long been a friend to us, and it is a pleasure that she is now finally receiving a species dedicated to her.

#### 3.1.4. *Aulonastus fringillus* Skoracki, 2011

Diagnosis. Females. Total body length 450–480. Infracapitulum sparsely punctate. Each medial branch of peritremes with 2 chambers, each lateral branch with 4–5 chambers. Stylophore 120–125 long. Propodonotal shield punctate. Setae *c1* 1.2–1.3 times longer than *se*. Length ratio of setae *d2*:*c1* 1:1.9–2. Hysteronotal shield fused to pygidial shield, apunctate. Setae *f2* twice as long as *f1*. Setae *h2* 6.6–7.6 times longer than *f2*. Aggenital setae *ag1* and *ag2* subequal in length. All coxal fields apunctate. Fan-like tarsal setae III–IV *p*′ and *p*″ of legs III and IV with six tines. Lengths of setae: *se* 120–125, *c1* 140–165, *c2* 90–100, *d2* 80–90, *f1* 20, *f2* 40–50, *h1* 15–20, *h2* 305–330, *ag1* 90–95, *ag2* 80–90, *ag3* 125–135.

##### Host and Distribution

Monoxenous parasite associated with Common Chaffinch *Fringilla coelebs* Linnaeus (Fringillinae) in Poland [42].

##### Remarks

The mite material collected from *Linaria cannabina* was previously misidentified as *Aulonastus fringillus* [51]. However, re-examination of the specimens from this host clearly indicates that the material belongs to *Aulonastus loxius*. Consequently, *Linaria cannabina* should be excluded from the host spectrum of *Aulonastus fringillus* and instead included in the host spectrum of *Aulonastus loxius* (see below).

#### 3.1.5. *Aulonastus loxius* Skoracki, 2011

Diagnosis. Females. Total body length 400–445. Infracapitulum apunctate. Each medial branch of peritremes with 2 chambers, each lateral branch with 3–4 chambers. Stylophore 120–130 long. Propodonotal shield punctate. Setae *c1* 1.4 times longer than *se*. Length ratio of setae *d2*:*c1* 1:1.3. Hysteronotal shield fused to pygidial shield, apunctate. Setae *f2* about four times longer than *f1*. Setae *h2* 2.7–3.2 times longer than *f2*. Setae *ag1* 1.2 times longer than *ag2*. Coxal fields IV sparsely punctate. Fan-like tarsal setae III–IV *p*′ and *p*″ of III–IV legs with 6–7 tines. Lengths of setae: *se* 145–170, *c1* 170–205, *c2* 120–155, *d2* 130–145, *f1* 25, *f2* 80–105, *h1* 20–25, *h2* 255–325, *ag1* 70–80, *ag2* 60–70, *ag3* 100–120.

##### Hosts and Distribution

Mesostenoxenous parasite associated with birds of the subfamily Carduelinae: Red Crossbill *Loxia curvirostra* Linnaeus in Poland [42], and Eurasian Linnet *Linaria cannabina* (Linnaeus) (Carduelinae) in Germany ([51], current paper).

##### New Material Examined

Ex *Linaria cannabina*; Germany: Darmstadt, Griesheim, March 1972, coll. H. Friemann—2 females deposited in the AMU (reg. no. AMU-MS 25-0516-001).

#### 3.1.6. *Aulonastus neotropicalis* Sikora, Unsoeld, Melzer, Friedrich, Hromada and Skoracki, 2025

Diagnosis. Females. Total body length 460–475. Infracapitulum apunctate. Each medial branch of peritremes with two chambers; each lateral branch with five chambers. Stylophore 130–140 long. Propodonotal shield apunctate. Setae *c1* 1.2 times longer than *se*. Length ratio of setae *d2*:*c1* 1:1.2. Hysteronotal shield fused to pygidial shield, apunctate. Setae *f2* 3–3.5 times longer than *f1*. Setae *h2* 4–4.4 times longer than *f2*. Aggenital setae *ag1* 1.3–1.7 longer than *ag2*. All coxal fields apunctate. Fan-like setae *p*′ and *p*″ of legs III and IV with 5–6 tines. Lengths of setae: *se* 160–180, *c1* 190–210, *c2* 150–165, *d2* 130–165, *f1* 20–25, *f2* 70–80, *h1* 20–25, *h2* 270–320, *ag1* 65–85, *ag2* 40–50, *ag3* 105–110.

##### Hosts and Distribution

Mesostenoxenous parasite associated with birds of the subfamily Euphoninae: Blue-naped Chlorophonia *Chlorophonia cyanea* (Thunberg) in Venezuela and Bolivia; Lesser Antillean Euphonia *Chlorophonia flavifrons* (Sparrman) in Guadeloupe (Lesser Antillean Creole); Golden-rumped Euphonia *Chlorophonia cyanocephala* (Vieillot), Scrub Euphonia *Euphonia affinis* (Lesson), Velvet-fronted Euphonia *Euphonia concinna* Sclater, all three in Colombia; Yellow-crowned Euphonia *Euphonia luteicapilla* (Cabanis) in Panama; Tawny-capped Euphonia *Euphonia anneae* Cassin in Costa Rica; Orange-bellied Euphonia *Euphonia xanthogaster* Sundevall in Peru; White-lored Euphonia *Euphonia chrysopasta* Sclater and Salvin in Venezuela, and Golden-sided Euphonia *Euphonia cayennensis* (Gmelin) in French Guiana [55].

#### 3.1.7. *Aulonastus ritzerfeldi* sp. n. (Figure 3)

Description. Female, holotype. Total body length 510 (480–540 in six paratypes). Gnathostoma. Infracapitulum apunctate. Hypostomal apex smooth, without protuberances. Stylophore rounded posteriorly, 140 (140–145) long, exposed portion of stylophore apunctate, 105 (100–105) long. Movable cheliceral digit 95 (95) long. Medial branch of peritremes with two chambers, each lateral branch with four delicately striated chambers. Idiosoma. Propodonotal shield weakly sclerotised, 115 (115–120) long, 60 (60–65) wide on level of setae *si*, rectangular in shape, apunctate, bearing bases of setae *ve*, *si* and *c1*, bases of setae *se* situated on or near this shield, bases of setae *c2* out of this shield. Setae *se* and *c1* situated at same transverse level, or setae *se* slightly anterior to level of setae *c1*. Setae *ve* and *si* short and subequal in length. Setae *c1* 1.4–1.5 times longer than *se*. Hysteronotal shield apunctate, fused to pygidial shield, anterior margin reach level of setal bases *d1*, hysteronotal shield 170 long and 40 wide on level of setae *e2*. Setae *d2* distinctly (9–13 times) longer than *d1* and *e2* but about 2 times shorter than *c1*. Setae *f2* 3 times longer than *f1*. Setae *f1* and *h1* subequal in length. Both pairs of genital setae equal in length. All coxal fields weakly sclerotised and sparsely punctate. Aggenital setae *ag1* and *ag2* subequal in length. Genital plate absent. Legs. Fan-like tarsal setae *p*′ and *p*″ of legs III and IV with 7–8 tines. Lengths of setae: *ve* 20 (15–20), *si* 20 (15–20), *se* 125 (120–140), *c1* 190 (170–190), *c2* 115 (115–125), *d1* 10 (10), *d2* 80 (70–80), *e2* 15 (10–15), *f1* (20–25), *f2* 60 (60–70), *h1* 20 (20–25), *h2* (305–340), *ps1* 15 (15), *g1* and *g2* 25 (25), *ag1* 95 (85–95), *ag2* 100 (95–105), *ag3* 130 (135–150), *3b* 25 (20–30), *3c* 60 (55–60), *4b* 25 (25), *4c* (60–65), *l*′*RIII* 30 (30–35), *l*′*RIV* (25–30).

**Figure 3:**
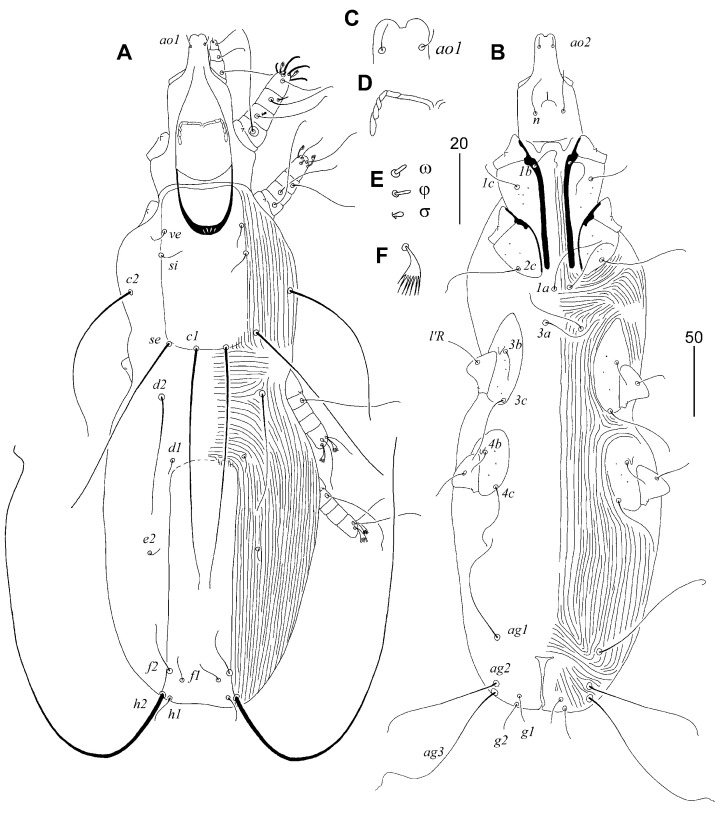
*Aulonastus ritzerfeldi* sp. n., female: (**A**) dorsal view; (**B**) ventral view; (**C**) hypostomal apex; (**D**) peritreme; (**E**) solenidia of leg I; (**F**) fan-like tarsal seta III. Scale bars: (**A**,**B**) = 50 µm, (**C**–**F**) = 20 µm.

Male. Not found.

##### Type Material

Female holotype and six female paratypes from Asian Rosy-Finch *Leucosticte arctoa* (Pallas) (Carduelinae) (host reg. no. ZSM 28.1041, male); Japan: Kobe, 16 April 1915, coll. J. Gengler.

##### Type Material Deposition

Holotype and most paratypes are deposited in the SNSB-ZSM (reg. no. ZSMA20250028), except two female paratypes in the AMU (reg. no. MS 24-1025-094).

##### Etymology

This species is named in honour of our colleague and good friend Marc Ritzerfeld from the SNSB, Bavarian State Collection for Zoology, for his great expertise and tireless diligence in recording and maintaining natural history collection data.

##### Differential Diagnosis

*Aulonastus ritzerfeldi* sp. n. is morphologically similar to *A. fringillus* Skoracki, 2011. In females of both species, each medial and lateral branch of the peritremes has a similar number of chambers (two and four, respectively); the movable cheliceral digit is 90 µm long; setae *se* and *c1* are situated at the same transverse level; setae *c1* are approximately twice as long as *d2*; aggenital setae *ag1* and *ag2* are subequal in length; the genital plate is absent; and both pairs of genital setae are subequal in length. This new species differs from *A. fringillus* in the following features: in females of *A. ritzerfeldi* sp. n., the infracapitulum and the propodonotal shield are apunctate; the length of the stylophore is 140–145 µm; the propodonotal shield is rectangular in shape; the anterior margin of the hysteronotal shield reach the level of the setal bases *d1*; setae *f2* are approximately three times longer than *f1*; all coxal fields are sparsely punctate; fan-like setae *p*′ and *p*″ of legs III and IV have 7–8 tines, and the lengths of setae are *c1* and *f2* are 170–190 µm and 60–70 µm, respectively. In contrast, in females of *A. fringillus*, the infracapitulum and the propodonotal shield are punctate; the stylophore is 120–125 µm long; the propodonotal shield has a concave anterior margin; the anterior margin of the hysteronotal shield not reach the level of the setal bases *d1*; setae *f2* are about twice as long as *f1*; all coxal fields are apunctate; fan-like setae *p*′ and *p*″ of legs III and IV have six tines, and the lengths of setae *c1* and *f2* are: 140–165 µm and 40–50 µm, respectively.

#### 3.1.8. *Syringophiloidus carpodaci* Bochkov and Apanaskevich, 2001

Diagnosis. Females. Total body length 690–745. Infracapitulum densely punctate. Each medial branch of peritremes with 7–8 chambers, each lateral branch with 8–9 chambers. Stylophore 155–160 long. Propodonotal shield punctate in anterior part. Length ratio of setae *vi*:*ve*:*si* 1:1.6–1.8:3. Propodonotal setae thin with discernible ornament. Hysteronotal shield apunctate, not fused to pygidial shield. Pygidial shield punctate in posterior part. Setae *d2* 1.2–1.3 times longer than *e2*. Length ratio of setae *ag1*:*ag2*:*ag3* 1:1:1.2. Setae *ps1* and *ps2* subequal in length. Genital plate weakly sclerotised. Coxal fields I–IV punctate. Fan-like setae *p*′ and *p*″ of legs III and IV with 7–8 tines. Lengths of setae: *vi* 40–45, *ve* 70–75, *si* 125–130, *se* 230, *c1* 220–230, *c2* 225, *d1* 140–180, *d2* 180–215, *e2* 140–175, *f1* and *h1* 40, *f2* 235–275, *h2* 300–320, *ps1* and *ps2* 25, *g1* and *g2* 40–45, *ag1* 170–185, *ag2* 180–215, *ag3* 225–230, *tc*′*III–IV* 35, *tc*″*III–IV* 65–70, *3b* 25, *3c* 100–110, *l*′*RIII* 35.

##### Hosts and Distribution

Oligoxenous parasite associated with birds of the genus *Carpodacus* (Carduelinae): Common Rosefinch *Carpodacus erythrinus* (Pallas) in Poland, Kazakhstan, Russia, and Nepal ([42,56], current paper), and Scarlet Finch *Carpodacus sipahi* (Hodgson) in Nepal [current paper].

##### New Material Examined

Ex *Carpodacus erythrinus* (host reg. no. ZSM 62.1712, male); Nepal: Khumjung, Khumbu, 7 August 1962, coll. unknown—eight females and four males deposited in the AMU (reg. no. AMU-MS 24-1025-065).

Ex *Carpodacus sipahi* (host reg. no. ZSM A.1246, male); Nepal: no other data—10 females and 4 males deposited in the AMU (reg. no. AMU-MS 24-1025-074).

#### 3.1.9. *Syringophiloidus coccothraustes* Skoracki, 2011

Diagnosis. Females. Total body length 680–720. Infracapitulum sparsely punctate. Each medial branch of peritremes with 2–3 chambers, each lateral branch with 8–9 chambers. Stylophore 140–145 long. Propodonotal shield sparsely punctate on whole surface. Length ratio of setae *vi*:*ve*:*si* 1:1:2–3. Setae *vi* and *ve* thin and smooth, other propodonotal setae with delicate ornament. Hysteronotal shield not fused to pygidial shield, sparsely punctate. Setae *d2* and *e2* subequal in length. Pygidial shield densely punctate posteriorly. Length ratio *ag1*:*ag2*:*ag3* 1:1:1–1.2. Setae *ps2* 1.7 times longer than *ps1*. Genital plate present. Coxal fields I–IV punctate. Fan-like setae *p*′ and *p*″ of legs III and IV with six tines. Lengths of setae: *vi* 25–35, *ve* 25–35, *si* 55–90, *se* 195–200, *c1* 190–205, *c2* 160–170, *d1* 145–160, *d2* 140–170, *e2* 140–150, *f1* 30, *f2* 200–230, *h1* 30, *h2* 305–330, *ps1* 12–15, *ps2* 20–25, *g1* 35–40, *g2* 35, *ag1* 130–160, *ag2* 140–155, *ag3* 145–175.

##### Host and Distribution

Monoxenous parasite associated with Hawfinch *Coccothraustes coccothraustes* (Linnaeus) (Carduelinae) in Poland [42].

#### 3.1.10. *Syringophiloidus klimovi* Skoracki and Bochkov, 2010

Diagnosis. Females. Total body length 680–705. Infracapitulum punctate. Each medial branch of peritremes with 2 chambers, each lateral branch with 6–7 chambers. Stylophore apunctate, 145 long. Propodonotal shield punctate anteriorly. Setae *vi*, *ve*, and *si* thin and smooth. Setae *se* and *c1* enlarged in basal part. Setae *vi* and *ve* subequal in length, both 1.8–2.2 times shorter than *si*. Hysteronotal shield absent, punctate area near bases of setae *d2* present. Small pygidial shield punctate and restricted to bases of setae *f1*, *f2*, *h1* and *h2*. Setae *h2* 1.5 times longer than *f2*. Setae *ag1* and *ag3* slightly (1.2–1.4 times) longer than *ag2*. Setae *ps1* and *ps2* subequal in length. Coxal fields I–IV punctate. Fan-like setae *p*′ and *p*″ of legs III and IV with 7–9 tines. Lengths of setae: *vi* 30–40, *ve* 30–40, *si* 70–75, *se* 140–150, *c1* 155–170, *c2* 130–150, *d1* 105, *d2* 50–55, *e2* 110, *f1* 30, *f2* 170–180, *h1* 30–35, *h2* 255, *ps1* and *ps2* 15–20, *g1* and *g2* 30, *ag1* 115–125, *ag2* 100, *ag3* 130–140.

##### Hosts and Distribution

Mesostenoxenous parasite associated with birds of the subfamily Carduelinae: European Greenfinch *Chloris chloris* (Linnaeus) in Kazakhstan and England [42,52] and Desert Finch *Rhodospiza obsoleta* (Lichtenstein) in Germany [51].

#### 3.1.11. *Syringophiloidus serini* Bochkov, Fain & Skoracki, 2004

Diagnosis. Females. Each medial branch of peritremes with 8 chambers, each lateral branch with 10–11 chambers. Propodonotal shield punctate. Setae *vi*, *ve*, and *si* enlarged basally and serrate. Hysterosomal plate fused to pygidial plate, punctate. Fan-like setae *p*′ and *p*″ of legs III and IV with 5–6 tines. Lengths of setae: *vi* 30–35, *ve* 40–55, *si* 115–130, *se* 210–240, *c2* 180–200, *c1* 215–220, *d1* 120–145, *f1* 17–22, *h1* 29–35, *d2* 150–165, *e2* 130–140, *f2* 165–180, *h2* 330–380, *ag1* 110–135, *ag2* 110–150, *ag3* 165–200, *g1* and *g2* ca. 25, *ps1* and *ps2* ca. 15.

##### Hosts and Distribution

Mesostenoxenous parasite associated with birds of the subfamily Carduelinae: Yellow-rumped Seedeater *Crithagra atrogularis* (Smith), Thick-billed Seedeater *Crithagra burtoni* (Gray), African Citril *Crithagra citrinelloides* (Rüppell), all from Tanzania [current paper], Yellow-fronted Canary *Crithagra mozambica* (Müller) in Central Africa [57], Twite *Linaria flavirostris* (Linnaeus) in Kyrgyzstan, and Yellow-crowned Canary *Serinus flavivertex* (Blanford) in Tanzania [current paper].

##### New Material Examined

Ex *Crithagra atrogularis* (host reg. no. ZSM 61.40); Tanzania: Manyara Region, Magugu, 21 July 1960, coll. v. Nagy—2 females deposited in the AMU (reg. no. AMU-MS 24-1025-113).

Ex *Crithagra burtoni* (host reg. no. ZSM 64.732, female); Tanzania: near Lake Tanganyika, 31 January 1951, coll. Th. Andersen—11 females and 2 males deposited in the AMU (reg. no. AMU-MS 24-1025-122).

Ex *Crithagra citrinelloides* (host reg. no. ZSM 60.1253, female); Tanzania: Arusha Region, Meru District, Usa River, 1200 m a.s.l., 19 April 1960, coll. v. Nagy—10 females deposited in the AMU (reg. no. AMU-MS 24-1025-110).

Ex *Linaria flavirostris* (host reg. no. ZSM 10.1744); Kyrgyzstan: Naryn Province, Naryn, 13 January 1910, coll. Merzbacher—five females and two males deposited in the AMU (reg. no. AMU-MS 24-1025-124).

Ex *Serinus flavivertex* (host reg. no. ZSM uncatalogued, male); Tanzania: Njombe District, 21 September 1950, coll. Th. Andersen—nine females and one male deposited in the AMU (reg. no. AMU-MS 24-1025-153).

#### 3.1.12. *Syringophiloidus stawarczyki* Skoracki, 2004

Diagnosis. Females. Total body length 605–695. Infracapitulum punctate. Each medial branch of peritremes with 2–3 chambers, each lateral branch with 11–12 chambers. Stylophore 170–195 long. Propodosomal shield punctate. Setae *vi* and *ve* thin, slightly serrate and subequal in length. Setae *si* variable in length but always longer than *vi* and *ve*. Hysteronotal shield not fused to pygidial shield, punctate, anterior margin reach above level of setal bases *d2*. Pygidial shield punctate. Setae *ps2* longer than *ps1*. Setae *ag1* and *ag2* subequal in length. All coxal field punctate. Fan-like setae *p*′ and *p*″ of legs III–IV with 7–8 tines. Lengths of setae: *vi* 15, *ve* 15–20, *si* variable 30–60, *se* 165–170, *c1* 195, *d1* 145–160, *d2* 115–125, *e2* 145–150, *f1* 25, *f2* 220, *h1* 25, *h2* 305, *ps1* 10, *ps2* 15–20, *ag1* 130–135, *ag2* 125–135, *ag3* 165.

##### Hosts and Distribution

Mesostenoxenous parasite associated with birds of the subfamily Euphoninae: Blue-naped Chlorophonia *Chlorophonia cyanea* (Thunberg) in Brazil and Colombia [55]; Golden-rumped Euphonia *Chlorophonia cyanocephala* (Vieillot) in Brazil, Paraguay and Colombia [55,58]; Purple-throated Euphonia *Euphonia chlorotica* (Linnaeus) in Paraguay; White-lored Euphonia *Euphonia chrysopasta* (Sclater & Salvin) in Venezuela and Bolivia; Velvet-fronted Euphonia *Euphonia concinna* (Sclater) in Colombia; Thick-billed Euphonia *Euphonia laniirostris* (d’Orbigny & Lafresnaye) in Venezuela, and Violaceous Euphonia *Euphonia violacea* (Linnaeus) in Trinidad and Tobago [55].

#### 3.1.13. *Syringophilopsis euphonicus* Sikora, Unsoeld, Melzer, Friedrich, Hromada and Skoracki, 2025

Diagnosis. Females. Total body length 950–1040. Infracapitulum apunctate. Hypostomal apex with one pair of small and sharp-ended protuberances. Stylophore 230–250 long. Each medial branch of peritremes with 2–3 chambers, each lateral branch with 12 chambers. Propodonotal shield sparsely punctate near bases of setae *ve* and *si*. Bases of setae *se* and *c1* situated at same transverse level. Bases of setae *c2* situated posterior to level of setal bases *si*. Length ratio of setae *vi*:*ve*:*si* 1:1.5–2:3.5–4. Hysteronotal shields absent. Pygidial shield apunctate. Setae *ag2* 3.8–4 times longer than *g1* and *g2*. Setae *ag1* and *ag2* long and subequal in length. Coxal fields III–IV densely punctate. Apodemes I and II fused in anterior part of apodemes II. Lengths of setae: *vi* 90–95, *ve* 140–180, *si* 315–355, *se* 335–390, *c1* 320–380, *c2* 325–350, *d1* 320–410, *d2* 320–360, *e2* 300–400, *f1* > 250, *f2* 400, *h1* 290–345, *h2* 415–470, *g1* and *g2* 35–50, *ag1* 195–295, *ag2* 190–235, *ag3* 230–300.

##### Hosts and Distribution

Oligoxenous parasite associated with birds of the subfamily Euphoninae: White-vented Euphonia *Euphonia minuta* (Cabanis) in Colombia; Orange-bellied Euphonia *Euphonia xanthogaster* (Sundevall) in Peru, and Trinidad Euphonia *Euphonia trinitatis* (Strickland) in Venezuela [55].

#### 3.1.14. *Syringophilopsis fringillae* (Fritsch, 1958)

Diagnosis. Females. Total body length 1200–1310. Infracapitulum apunctate. Hypostomal apex with one pair of short protuberances. Each medial branch of peritremes with 3–5 chambers, each lateral branch with 13–14 chambers. Stylophore 255 long. Propodonotal shield punctate near bases of setae *ve* and *si*. Length ratio of setae *vi*:*ve*:*si* 1:1.5:1.5–2. Setae *se* and *c1* situated at same transverse level. Hysteronotal shields apunctate. Small pygidial shield sparsely punctate. Setae *h1* 1.8–2 times shorter than *f1*, both shorter than *f2* and *h2*. Genital setae shorter than aggenital setae *ag1* and *ag3*. Length ratio of setae *g1*:*ag2* 1:2.8–3. Setae *ag1* 1.2 times longer than *ag2*. Coxal fields I–IV punctate. Fan-like setae *p*′ and *p*″ of legs III and IV with 15–17 tines. Apodemes I and II fused in anterior part of apodemes II. Lengths of setae: *vi* 205–215, *ve* 310–315, *si* 325–395, *se* 405–410, *c1* 425, *c2* 390–395, *d1* 425, *d2* 395, *e2* 455, *f1* 185–190, *f2* 470–505, *h1* 340–350, *h2* 470–505, *ps1* and *ps2* 55, *g1* and *g2* 90–100, *ag1* 370–395, *ag2* 285–325, *ag3* 395.

##### Hosts and Distribution

Oligoxenous parasite associated with birds of the subfamily Fringillinae: Common Chaffinch *Fringilla coelebs* Linnaeus in England, Germany, Poland, Slovakia, Russia and Kazakhstan [42,52,58,59,60,61,62], and Brambling *Fringilla montifringilla* Linnaeus in Germany [51].

#### 3.1.15. *Syringophilopsis kirgizorum* Bochkov, Mironov and Kravtsova, 2000

Diagnosis. Females. Total body length 900–1180. Infracapitulum apunctate. Hypostomal apex with one pair of short and sharp-ended protuberances. Each medial branch of peritremes with 3–4 chambers, each lateral branch with 9–11 chambers. Stylophore 240–245 long. Propodonotal shield punctate near bases of setae *ve* and *si*. Length ratio of setae *vi*:*ve*:*si* 1:2:3.5–4. Setae *se* and *c1* situated at same transverse level. Hysteronotal shields absent. Small pygidial shield punctate. Genital setae shorter than aggenital setae *ag1* and *ag3*. Length ratio of setae *g1*:*ag2* 1:2.5–3. Setae *ag1* 1.3 times longer than *ag2*. Coxal fields I–IV punctate. Fan-like setae *p*′ and *p*″ of legs III and IV with 11–13 tines. Apodemes I and II fused in middle part of apodemes II. Lengths of setae: *vi* 60, *ve* 90–130, *si* 195–220, *se* 240–295, *c1* 305–325, *c2* 255–295, *d1* 215–280, *d2* 215–275, *e2* 255–280, *f1* 80, *f2* 375, *h1* 80, *ps1* and *ps2* 45, *g1* and *g2* 60–70, *ag1* 200–220, *ag2* 160–180, *ag3* 300–370.

##### Hosts and Distribution

Mesostenoxenous parasite associated with birds of the subfamily Carduelinae: European Goldfinch *Carduelis carduelis* (Linnaeus) in Poland and Russia [42,58]; European Greenfinch *Chloris chloris* (Linnaeus) in Germany, Poland, England, Jordan, and Kyrgyzstan [42,51,58,63,64,65]; Eurasian Linnet *Linaria cannabina* (Linnaeus) in Jordan [65]; Oriole Finch *Linurgus olivaceus* (Fraser) [66], and Desert Finch *Rhodospiza obsoleta* (Lichtenstein) in Kyrgyzstan [63].

#### 3.1.16. *Torotrogla cardueli* Bochkov and Mironov, 1999

Diagnosis. Females. Total body length 800–875. Hypostomal apex with one pair of medium-sized, blunt-ended and bill-like protuberances. Each medial branch of peritremes with 3–4 chambers, each lateral branch with 6–7 chambers. Propodonotal shield densely punctate on whole surface. Length ratio of setae *vi*:*ve*:*si* 1:1.3:3. Setae *c2* situated anterior to level of setae *se*. Hysteronotal shields punctate, bases of setae *d1* near these shields. Pygidial shield punctate. Setae *f1* and *h1* subequal in length. Coxal fields I–IV sparsely punctate. Fan-like setae *p*′ and *p*″ of legs III and IV with 10–11 tines. Lengths of setae: *vi* 55–75, *ve* 75, *si* 165–185, *se* 175, *c1* 215, *c2* 175, *d1* 150–170, *d2* 140, *e2* 145–170, *h1* 70–75, *h2* 440–490, *f1* 75–85, *f2* 375.

##### Hosts and Distribution

Mesostenoxenous parasite associated with birds of the subfamily Carduelinae: European Goldfinch *Carduelis carduelis* Linnaeus in Poland; Common Linnet *Linaria cannabina* (Linnaeus) in Poland and Slovakia; Red Crossbill *Loxia curvirostra* Linnaeus in Poland; White-winged Crossbill *Loxia leucoptera* Gmelin in Poland; Parrot Crossbill *Loxia pytyopsittacus* Borkhausen in Finland; Atlantic Canary *Serinus canaria* (Linnaeus) in Europe [42]; Fire-fronted Serin *Serinus pusillus* (Pallas) in Kyrgyzstan [current paper], and Eurasian Siskin *Spinus spinus* (Linnaeus) in Poland, Slovakia, and Russia [42,67].

##### New Material Examined

Ex *Serinus pusillus* (host reg. no. ZSM 17.3034, male); Kyrgyzstan: Naryn Province, Naryn, 9 April 1910, coll. Akulin—1 female and 10 males deposited in the AMU (reg. no. AMU-MS 24-1025-152).

#### 3.1.17. *Torotrogla coccothraustes* Bochkov, Flannery and Spicer, 2009

Diagnosis. Females. Total body length 1360–1400. Hypostomal apex with one pair of slim and elongated protuberances. Each lateral branch of peritremes with six chambers, each medial branch with three chambers. Propodonotal shield concave on anterior and posterior margins, apunctate. Length ratio of setae *vi*:*ve*:*si* 1:1.4:1.9. Setae *c2* situated anterior to level of setae *se*. Hysteronotal shields punctate, bases of setae *d1* situated on these shields. Pygidial shield punctate. Setae *f1* and *h1* subequal in length. Coxal fields I–IV sparsely punctate. Fan-like setae *p*′ and *p*″ of legs III and IV with 6–9 tines. Lengths of setae: *vi* 135–140, *ve* 150–165, *si* 245–270, *se* 285–300, *c1* 260–280, *c2* 300–330, *d1* 280–320, *d2* 280–320, *e2* 280–320, *h1* 120–140, *h2* 380–420, *f1* 110–125, *f2* 400–420, *ps1* and *ps2* 40, *g1* and *g2* 50.

##### Host and Distribution

Monoxenous parasite associated with Evening Grosbeak *Coccothraustes vespertinus* (Cooper) in the United States [68].

#### 3.1.18. *Torotrogla gaudi* Bochkov and Mironov, 1998

Diagnosis. Females. Total body length 800–875. Hypostomal apex with one pair of blunt-ended protuberances. Each medial branch of peritremes with 3–4 chambers, each lateral branch with 8–10 chambers. Propodonotal shield sparsely punctate near lateral margins. Length ratio of setae *vi*:*ve*:*si* 1:1.6–2:3.6–4. Setae *c2* situated anterior to level of setae *se*. Hysteronotal shields punctate, bases of setae *d1* near this shields. Pygidial shield apunctate. Coxal fields I–IV punctate. Fan-like setae *p*′ and *p*″ of legs III and IV with 10–12 tines. Lengths of setae: *vi* 65, *ve* 105–135, *si* 220–260, *se* 225–245, *c1* 320, *c2* 215–270, *d1* 205–275, *d2* 175, *e2* 230–250, *f1* 105–165, *f2* 610, *h1* 210–295, *h2* 500–645, *ps1* and *ps2* 35–45, *g1* 60, *g2* 40–45.

##### Hosts and Distribution

Mesostenoxenous parasite associated with birds of the subfamily Fringillinae: Common Chaffinch *Fringilla coelebs* Linnaeus in Poland and Russia [42,60], and Brambling *Fringilla montifringilla* Linnaeus in Poland and Slovakia [42,69].

##### Remark

The species *Torotrogla gaudi* was also previously recorded from the Eurasian Bullfinch *Pyrrhula pyrrhula* (Linnaeus) in Poland [69]. However, re-examination of the available material (3 females ex *P. pyrrhula* from Poland, West Pomeranian Voivodeship, near Gryfino, June 2001, coll. G. Kiljan, deposited in the AMU (reg. no. AMU MS 25-0706-001)) revealed that the mites collected from this host cannot be assigned to *T. gaudi*, as they exhibit markedly longer setae *vi*, *ve*, and *h1*, and probably represent a new, undescribed species. Nevertheless, the poor preservation of the available specimens precludes their unambiguous identification. Therefore, we propose excluding the Eurasian Bullfinch from the host spectrum of *T. gaudi* until new, well-preserved specimens from this host can be obtained.

#### 3.1.19. *Torotrogla enucleator* sp. n. (Figure 4)

Description. Female, holotype. Total body length 1140 (1080–1150 in four paratypes). Gnathosoma. Hypostomal apex with one pair of medium-sized and blunt-ended protuberances. Each medial branch of peritremes with 4–5 chambers, each lateral branch with 9–10 chambers. Stylophore constricted posteriorly, 370 (300–390) long, exposed portion of stylophore apunctate, 300 (250–330) long. Idiosoma. Propodonotal shield rectangular in shape, cleft on anterior margin, punctate laterally, width at level of setae *si* 160 (150–160). Bases of setae se situated anterior to level of setal bases *c1*, *c2* anterior to *se*. Length ratio of setae *vi*:*ve*:*si* 1:1.5–1.7:2.4–2.6. Pair of hysteronotal shields situated in close proximity to each other, punctate, bearing bases of setae *d1* on anterior margin. Pygidial shield apunctate, with rounded anterior margin, 180 long. Setae *h1* distinctly longer than *f1*. Setae *g1* slightly (1.2–1.3 times) longer than *g2*. Setae *ps1* and *ps2* subequal in length. Coxal fields I–IV punctate. Setae *3c* 1.8 times longer than *3b*. Legs. Fan-like setae *p*′ and *p*″ of legs III and IV with 6–9 tines. Lengths of setae: *vi* 105 (105–110), *ve* 165 (165–180), *si* 260 (255–270), *se* 260 (260–270), *c1* (260–305), *c2* (280–300), *d1* 215 (190–220), *d2* 190 (190–210), *e2* 200 (190–220), *f1* (80–90), *f2* 430 (400–450), *h1* (445–495), *h2* 470 (460–530), *ps1* 60 (50–60), *ps2* 55 (45–55), *g1* 80 (80–90), *g2* 60 (60–65), *l’RI* 50 (50), *l’RII* 55 (55–60), *l’RIII* (75–90), *l’RIV* 75 (70–75), *3b* 95 (85–95), *3c* 170 (170–185), all aggenital setae (*ag*) longer than 300.

Male. Not found.

##### Type Material

Female holotype and four female paratypes from Pine Grosbeak *Pinicola enucleator* (Linnaeus) (host reg. no. ZSM uncatalogued, female); Japan: Kuril Islands, Iturup, 28 August 1900, coll. Haberer.

##### Type Material Deposition

Holotype and two female paratypes are deposited in the SNSB-ZSM (reg. no. ZSMA20250029), except two female and one male paratypes in the AMU (reg. no. AMU MS 24-1025-082).

##### Additional Material

Ex Plain Mountain-Finch *Leucosticte nemoricola* (Hodgson) (host reg. no. ZSM 17.4752); Kyrgyzstan: Tian Shan, vicinity of Naryn, 2 February 1910, coll. Neschiwjow—1 female deposited in the AMU (reg. no. AMU MS 24-1025-093).

**Figure 4:**
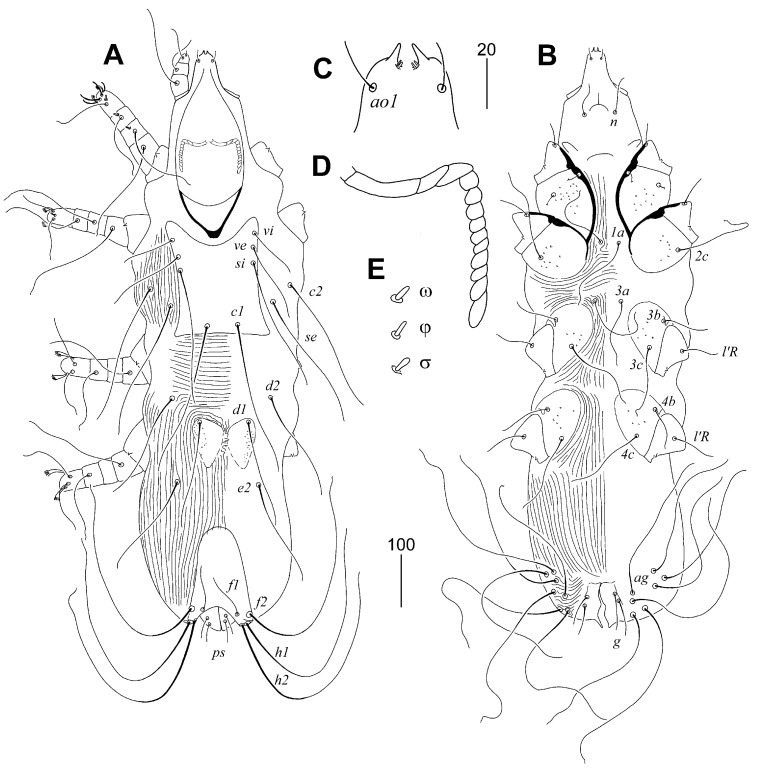
*Torotrogla enucleator* sp. n., female: (**A**) dorsal view; (**B**) ventral view; (**C**) hypostomal apex; (**D**) peritreme; (**E**) solenidia of leg I. Scale bars: (**A**,**B**) = 50 µm, (**C**–**E**) = 20 µm.

##### Differential Diagnosis

*Torotrogla enucleator* sp. n. is morphologically similar to *T. gaudi* Bochkov and Mironov, 1998. In females of both species, the hypostomal apex bears a pair of medium-sized and blunt-ended protuberances; each medial branch of the peritremes has 3–4 chambers, and each lateral branch has 8–10 chambers; the propodonotal shield is rectangular in shape and sparsely punctate near the lateral margins; setae *h1* are distinctly longer than *f1*; the pygidial shield is apunctate, and coxal fields I–IV are punctate. This new species differs from *T. gaudi* in the following features: in females of *T. enucleator*, setae *h1* are 4–5 times longer than *f1*; the lengths of setae *vi* and *ve* are 105–110 µm and 165–180 µm, respectively, and fan-like setae *p*′ and *p*″ of legs III and IV have 6–9 tines. In contrast, in females of *T. gaudi*, setae *f1* are half the length of *h1*; the lengths of setae *vi* and *ve* are 65 µm and 105–135 µm, respectively, and fan-like setae *p*′ and *p*″ of legs III and IV have 10–12 tines.

##### Etymology

This species name is taken from the specific name of the type host species—*Pinicola enucleator*.

#### 3.1.20. *Torotrogla janhafti* sp. n. (Figure 5 and Figure 6)

Description. Female, holotype. Total body length 925 (830–950 in six paratypes). Gnathosoma. Hypostomal apex with one pair of medium-sized and blunt-ended protuberances. Each medial branch of peritremes with 4 chambers, each lateral branch with 7–10 chambers. Stylophore constricted posteriorly, 345 (300–345) long, exposed portion of stylophore apunctate, 285 (250–280) long. Idiosoma. Propodonotal shield rectangular in shape, apunctate, width at level of setae *si* 140 (135–145). Bases of setae *se* situated anterior to level of setal bases *c1*, bases *c2* anterior to *se*. Length ratio of setae *vi*:*ve*:*si* 1:1.5–1.6:3–4. Hysteronotal shields punctate, bearing bases of setae *d1* on anterior margin. Pygidial shield apunctate, with rounded anterior margin, 120 long. Setae *f1* and *h1* short and subequal in length. Genital and pseudanal setae subequal in length. Coxal fields I–IV apunctate. Setae *3c* about twice as long as *3b*. Legs. Fan-like setae *p*′ and *p*″ of legs III and IV with 7–8 tines. Lengths of setae: *vi* 75 (55–75), *ve* 100 (90–110), *si* 230 (205–230), *se* 240 (215–240), *c1* 295 (235–275), *c2* (215–250), *d1* 210 (210–220), *d2* 190 (190–225), *e2* 210 (205–230), *f1* 80 (80–100), *f2* 495 (490–505), *h1* (120–170), *h2* 530 (515–530), *ps1* and *ps2* 40 (30–40), *g1* 40 (40–45), *g2* 35 (30–35), *l’RIII* 65 (60–65), *l’RIV* 65 (60–65), *3b* 55 (55–65), *3c* 100 (100–125), all setae *ag* longer than 300.

**Figure 5:**
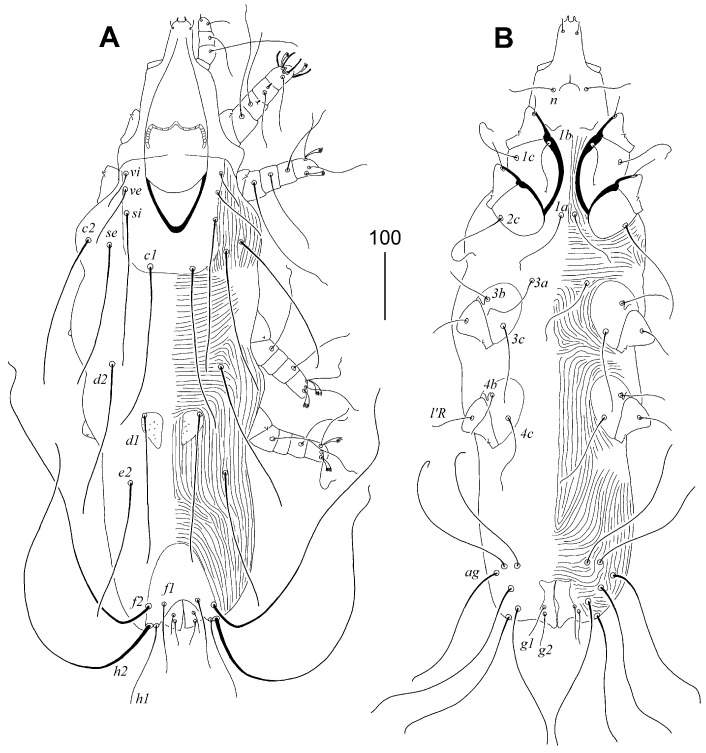
*Torotrogla janhafti* sp. n., female: (**A**) dorsal view; (**B**) ventral view.

Male. Total body length 690–710. Gnathosoma. Hypostomal apex with one pair of small and blunt-ended protuberances. Each medial branch of peritremes with 3–4 chambers, each lateral branch with 8–10 chambers. Stylophore constricted posteriorly, 230–240 long, exposed portion of stylophore apunctate, 190–200 long. Propodonotal shield with all margins concave, laterally punctate, width at level of setae *si* 125–130. Bases of setae *se* situated anterior to level of setal bases *c1*, *c2* situated anterior to *se*. Length ratio of setae *vi*:*ve*:*si* 1:2.8:2.5. Hysteronotal shield fused to pygidial shield, about 255 long, bearing bases of setae *d1*, *e2*, *f2* and *h2*, anterior margin not reaching bases of setae *d2*. Setae *d2* 1.3–1.6 times longer than *d1*. Coxal fields I–IV apunctate. Legs. Fan-like setae *p*′ and *p*″ of legs III and IV with six tines. Lengths of setae: *vi* 55, *ve* 70–85, *si* 130–140, *se* 125, *c1* 155, *c2* 125–150, *d1* 25, *d2* 20–40, *e2* 20, *f2* 55–60, *h2* 200–270, *l’RIII–IV* 40.

**Figure 6:**
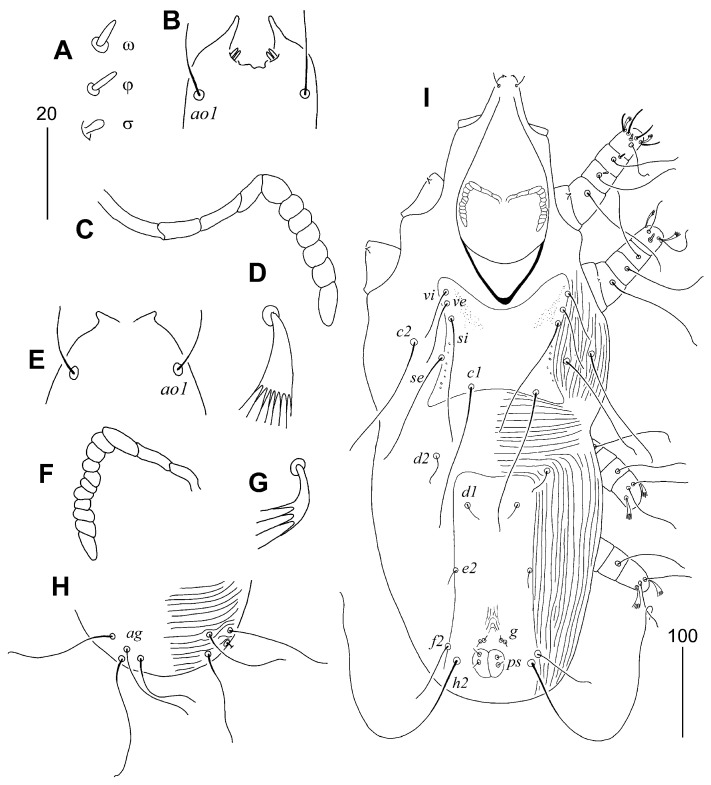
*Torotrogla janhafti* sp. n., female (**A**–**D**): (**A**) solenidia of leg I; (**B**) hypostomal apex; (**C**) peritreme; (**D**) fan-like seta p′III. Male (**E**–**I**): (**E**) hypostomal apex; (**F**) peritreme; (**G**) fan-like seta p′III; (**H**) opisthosoma in ventral view; (**I**) body in dorsal view. Scale bars: (**A**–**G**) = 20 µm, (**H**,**I**) = 50 µm.

##### Type Material

Female holotype and paratypes: six females and two males from Red-mantled Rosefinch *Carpodacus rhodochlamys* (Brandt) (host reg. no. ZSM 09.4519, male); Kyrgyzstan: Naryn, 21 March 1908, coll. Merzbacher.

##### Type Material Deposition

Holotype and most paratypes are deposited in the SNSB-ZSM (reg. no. ZSMA20250030), except two female and one male paratypes in the AMU (reg. no. AMU MS 24-1025-075).

##### Additional Material

Ex. Siberian Long-tailed Rosefinch *Carpodacus sibiricus* (Pallas) (host reg. no. ZSM 33.110, male); Japan: Hokkaido, Sapporo, 8 November 1906, coll. R. Fenk—eight females deposited in the ZSM (ZSMA20250031), eight females and one male in the AMU (reg. no. AMU MS 24-1025-079).

Ex Himalayan White-browed Rosefinch *Carpodacus thura* Bonaparte and Schlegel (host reg. no. ZSM 62.1733, male); Nepal: Khumbu Region, Khumjung, 14 June 1962, coll. unknown—two females and three males deposited in the AMU (reg. no. AMU MS 24-1025-080).

Ex Sinai Rosefinch *Carpodacus synoicus* (Temminck) (host reg. no. ZSM 1910/2, female); Egypt: Sinai, Wadi Debbet, 3 April 1909, coll. Schloesser—two females and two males deposited in the AMU (reg. no. MS 24-1025-076).

##### Differential Diagnosis

*Torotrogla janhafti* sp. n. is morphologically most similar to *T. cardueli* Bochkov and Mironov, 1999, in having a hypostomal apex with a pair of medium-sized blunt-ended protuberances, medial peritremal branches with 3–4 chambers, lateral branches with 6–7 chambers, setae *c2* situated anterior to *se*, punctate hysteronotal shields, and setae *f1* and *h1* subequal in length. The new species differs from *T. cardueli* in the following features: in females of *T. janhafti*, the propodonotal shield and coxal fields I–IV are apunctate, the fan-like setae *p*′ and *p*″ of legs III and IV have 7–8 tines, the length ratio of setae *vi:ve* is 1:1.5–1.6, and the lengths of setae *si*, *se*, *d2*, and *f2* are 205–230 µm, 215–240 µm, 190–225 µm, and 490–505 µm, respectively; in males, setae *vi* and *ve* measure 55 µm and 70–85 µm, respectively, the coxal fields I–IV are apunctate, and the fan-like setae *p*′ and *p*″ of legs III and IV have six tines. In contrast, in females of *T. cardueli*, the propodonotal shield and coxal fields I–IV are densely punctate, the fan-like setae *p*′ and *p*″ of legs III and IV have 10–11 tines, the length ratio of setae *vi:ve* is 1:1.3, and the lengths of setae *si*, *se*, *d2*, and *f2* are 165–185 µm, 175 µm, 140 µm, and 375 µm, respectively; in males, setae *vi* and *ve* measure 20 µm and 45 µm, respectively, the coxal fields I–IV are punctate, and the fan-like setae *p*′ and *p*″ of legs III and IV have 8–9 tines.

##### Etymology

The species is named in honour of the German nature and wildlife filmmaker Jan Michael Haft for his great work in producing particularly extraordinary and impressive nature documentaries.

### 3.2. Summarised Diversity of Syringophilinae Mites Associated with Fringillidae Birds

To date, representatives of the subfamily Syringophilinae have been recorded from numerous finch genera across all major zoogeographic regions inhabited by this bird family. The following table summarises the current knowledge on their species richness, host associations, and distribution, integrating both previously published records and new data from the present study (Table 1).

### 3.3. Key to Syringophilinae Species Associated with Birds of the Family Fringillidae

1.Propodonotal region with 5 pairs of setae (*vi* absent)—*Aulonastus*2Propodonotal region with 6 pairs of setae (*vi* present)52.Setae *f2* twice as long as *f1*
*Aulonastus fringillus*
Setae *f2* 3–4 times longer than *f1*33.Propodonotal shield punctate
*Aulonastus loxius*
Propodonotal shield apunctate44.Setae *c1* twice longer than *d2**Aulonastus ritzerfeldi* sp. n.Setae *c1* 1.2 times longer than *d2*
*Aulonastus neotropicalis*
5.Leg setae *dGII* absent—*Syringophiloidus*6Leg setae *dGII* present106.Medial branch of peritremes with 8 chambers7Medial branch of peritremes with 2–3 chambers87.All propodonotal setae thick and enlarged basally. Hysteronotal shield punctate
*Syringophiloidus serini*
All propodonotal setae thin and hair-like. Hysteronotal shield apunctate
*Syringophiloidus carpodaci*
8.Setae *d1* and *e2* twice as long as *d2*
*Syringophiloidus klimovi*
Setae *d1*, *d2*, and *e2* subequal in length99.Lateral branch of peritreme with 8–9 chambers. Infracapitulum sparsely punctate. Length of stylophore 140–145 μm
*Syringophiloidus coccothraustes*
Lateral branch of peritreme with 11–12 chambers. Infracapitulum densely punctate. Length of stylophore 170–195 μm
*Syringophiloidus stawarczyki*
10.Aggenital setal series with more than 4 pairs—*Torotrogla*11Aggenital setal series with 3 pairs1511.Total body length 1360–1400 μm
*Torotrogla coccothraustes*
Total body length less than 1100 μm1212.Setae *h1* distinctly longer than *f1*13Setae *f1* and *h1* subequal in length1413.Setae *h1* 210–295 μm long
*Torotrogla gaudi*
Setae *h1* 445–495 μm long*Torotrogla enucleator* sp. n.14.Propodonotal shield and coxal fields I–IV apunctate*Torotrogla janhafti* sp. n.Propodonotal shield and coxal fields I–IV densely punctate
*Torotrogla cardueli*
15.Apodemes I parallel and not fused to apodemes II—*Aulobia*16Apodemes I divergent and fused to apodemes II—*Syringophilopsis*1816.Each lateral branch of peritremes with 18–20 chambers
*Aulobia cardueli*
Each lateral branch of peritremes with 10–13 chambers1717.Hysteronotal shield absent*Aulobia ruppelae* sp. n.Hysteronotal shield present
*Aulobia leucostictus*
18.Hysteronotal shield absent19Hysteronotal shield present
*Syringophilopsis fringillae*
19.Setae *si* 315–355 μm long. Pygidial shield apunctate
*Syringophilopsis euphonicus*
Setae *si* 195–220 μm long. Pygidial shield punctate
*Syringophilopsis kirgizorum*


## 4. Discussion

**Figure 7:**
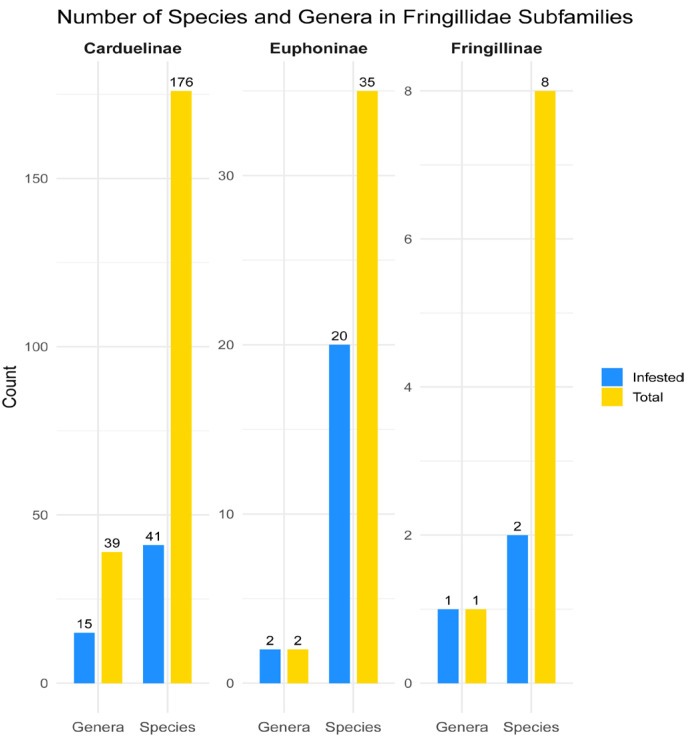
Representation of quill mite infestation across species and genera of Fringillidae subfamilies; the blue bars represent the number of host species/genera infested with quill mites, and the yellow bars indicate the total number of extant species within each subfamily.

The family Fringillidae comprises 236 species grouped into 49 genera [26], of which 17 species and 7 genera are considered extinct [43]. Considering only extant taxa, the family currently comprises 219 species belonging to 42 genera in 3 subfamilies: Carduelinae (176 species in 39 genera), Euphoninae (35 species in 2 genera), and Fringillinae (8 species in 1 genus). The global diversity of quill mites of the subfamily Syringophilinae, associated with the Fringillidae, currently comprises 20 species belonging to 5 genera. They have so far been recorded from 51 bird species, representing 23% of all extant Fringillidae species. These infested hosts belong to 18 genera, accounting for 43% of all fringillid genera, and span all 3 subfamilies (100%) (Figure 7). While the subfamilies Fringillinae and Euphoninae have been fully surveyed at the genus level, only 39% (15 out of 39 genera) of Carduelinae genera have been examined for quill mites. The following 24 genera in Carduelinae remain unexplored, many of which are monotypic or contain only a few species: *Agraphospiza* (1 species), *Bucanetes* (2), *Callacanthis* (1), *Chlorodrepanis* (3), *Chrysocorythus* (2), *Drepanis* (1), *Eophona* (2), *Haemorhous* (3), *Hemignathus* (1), *Himatione* (1), *Loxioides* (1), *Loxops* (3), *Magumma* (1), *Mycerobas* (4), *Oreomystis* (1), *Palmeria* (1), *Paroreomyza* (2), *Procarduelis* (1), *Psittirostra* (1), *Pseudonestor* (1), *Pyrrhoplectes* (1), *Rhodopechys* (1), *Rhynchostruthus* (3), and *Telespiza* (2). The number of unexamined genera within Carduelinae suggests that many new species of syringophilines may still await discovery and description. However, given that the major phylogenetic lineages of Fringillidae have already been studied, we do not expect the presence of additional quill mite genera beyond the five reported in this work.

**Figure 8:**
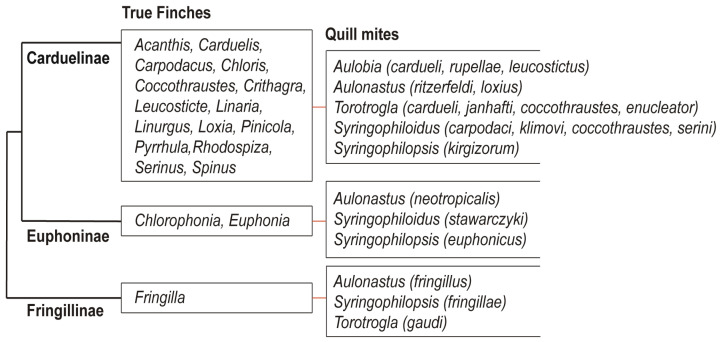
Phylogeny of the family Fringillidae on the subfamily level (after Zuccon et al. [33]) with the records of quill mites.

The present-day structure of the Fringillidae family is the result of decades of anatomical research [70,71,72,73] and recent advances in molecular phylogenetics [27,28,29,30,31,32,33,34]. Traditionally, the True Finches have been divided into three subfamilies: Fringillinae, which represents the most basal lineage; Euphoninae, the sister group to Carduelinae; and Carduelinae, the most derived and speciose clade [33,34] (Figure 8). The split between the Fringillidae and their sister family, the Emberizidae, is estimated to have occurred around 16.8 million years ago (mya) [34,74]. Likely, syringophilid mites were already associated with the common ancestor of these two lineages, and the Fringillidae inherited this parasitic association after the divergence. The diversification of quill mites in Fringillidae likely occurred later, in parallel with the radiation of their avian hosts. Among the three subfamilies, Carduelinae exhibits the highest diversity of associated syringophiline mites, hosting all five recorded genera: *Aulobia*, *Aulonastus*, *Syringophiloidus*, *Syringophilopsis*, and *Torotrogla*. In contrast, Euphoninae and Fringillinae each harbour only three genera of quill mites. This pattern may reflect both historical loss and limited opportunity for host–parasite co-diversification within the less speciose lineages. We hypothesise that the ancestral syringophiline community associated with the early Fringillidae comprised all five genera listed above. Following the divergence of Fringillinae around 14.6 mya, only *Aulonastus*, *Syringophilopsis*, and *Torotrogla* were retained within that clade. Similarly, the later split between Euphoninae and Carduelinae, estimated at 13.8 mya [34,74], led to the persistence of *Aulonastus* and *Syringophilopsis* in Euphoninae, but with the apparent loss of *Torotrogla* and the retention of *Syringophiloidus* [55]. Only Carduelinae, likely due to its extensive radiation, retained representatives of all five genera. A plausible explanation for the reduced diversity of quill mite genera observed in Fringillinae and Euphoninae lies in the limited taxonomic diversity of these host lineages. Euphoninae currently comprises just 35 species in 2 genera, while Fringillinae includes only 8 species in a single genus. In contrast, Carduelinae is markedly more diverse, with 176 species across 39 genera, offering greater opportunity for parasite speciation and persistence. This clear link between host taxonomic diversity and parasite genus richness suggests that the evolutionary and taxonomic structure of host lineages plays a pivotal role in shaping the composition and complexity of their parasite assemblages. This observation is consistent with Eichler’s rule, which posits a positive correlation between host and parasite diversity in coevolutionary systems.

For a long time, quill mites of the family Syringophilidae were considered to be predominantly monoxenous parasites or, at most, oligoxenous (parasitising hosts belonging to a single genus) [42,49]. However, increasingly frequent studies on the distribution of quill mites across entire bird families or orders indicate that these mites exhibit greater host diversity, suggesting a more substantial presence of oligoxenous or mesostenoxenous species, which infest hosts belonging to closely related genera [20,21,22,23,24,25]. In our study (see Table 1), we observed that the number of monoxenous species amounts to five: *Aulonastus fringillus*, *A. ritzerfeldi* sp. n., *Aulobia leucostictus*, *Syringophiloidus coccothraustes*, and *Torotrogla coccothraustes*—representing 25% of the total fauna. We identified six oligoxenous species—*Syringophiloidus carpodaci* associated with birds of the genus *Carpodacus*; *Syringophilopsis euphonicus* parasitising birds of the genus *Euphonia*; *Syringophilopsis fringillae* and *Torotrogla gaudi* from *Fringilla*; and *T. janhafti* and *Aulobia ruppellae* sp. n., both from *Carpodacus*—which make up 30% of all species. The largest proportion consists of mesostenoxenous species (45%, nine species), including *Aulobia cardueli* (associated with *Acanthis*, *Carduelis*, *Linaria*, *Spinus*), *Aulonastus loxius* (*Loxia*, *Linaria*), *A. neotropicalis* (*Chlorophonia*, *Euphonia*), *Syringophiloidus klimovi* (*Chloris*, *Rhodospiza*), *S. serini* (*Crithagra*, *Linaria*, *Serinus*), *S. stawarczyki* (*Chlorophonia*, *Euphonia*), *Syringophilopsis kirgizorum* (*Carduelis*, *Chloris*, *Linaria*, *Linurgus*, *Rhodospiza*), *Torotrogla cardueli* (*Carduelis*, *Linaria*, *Loxia*, *Serinus*, *Spinus*), and *T. enucleator* (*Pinicola*, *Leucosticte*). Importantly, no parasite species in our dataset was found to inhabit hosts from different subfamilies of Fringillidae, suggesting a strong phylogenetic constraint in host exploitation. The observed ratio of monoxenous (25%) to non-monoxenous (75%) species may reflect a general pattern in syringophilid mites associated with taxonomically diverse avian groups. This distribution supports the hypothesis of close co-phylogenetic relationships between parasites and their hosts. The presence of specific mite species on phylogenetically related hosts suggests a long-term evolutionary association within particular avian lineages.

**Figure 9:**
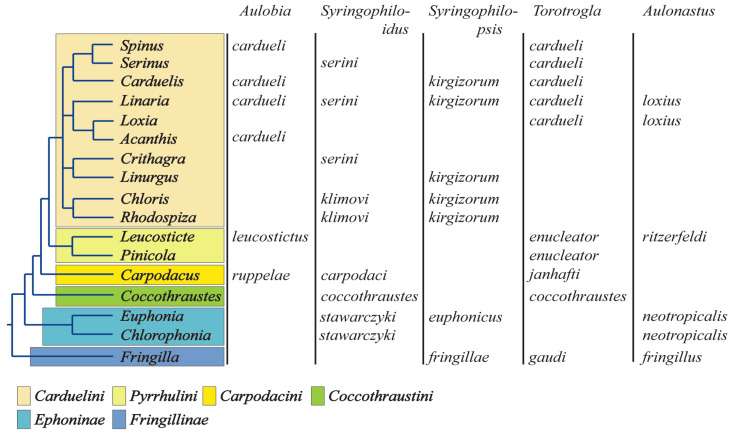
Phylogeny of the family Fringillidae (after Zuccon et al. [33], modified) with the records of quill mite genera and species.

The exploration of host–parasite cospeciation has intrigued scientists for over a century [75]. Given the deep evolutionary ties between parasites and their hosts, early researchers often considered parasites as extensions of their host phenotypes, anticipating that parasite phylogenies would closely mirror those of their hosts, an assumption encapsulated in Fahrenholz’s Rule [76]. However, it has become increasingly clear that the evolutionary histories of hosts and parasites are far more complex, with a range of events capable of obscuring or disrupting strict co-phylogenetic patterns [77,78,79]. Based on the results of our study, several key observations can be made. First, quill mites associated with the family Fringillidae do not exhibit a random host distribution, a characteristic expected of obligate parasites. Instead, individual mite species are typically restricted to either a single host species or a group of closely related hosts, fitting the definitions of oligoxenous or mesostenoxenous parasites, respectively (Figure 9). The presence of the same quill mite species on representatives of hosts belonging to phylogenetically closely related genera may result from delayed parasite speciation relative to host speciation, a phenomenon referred to as retardation, which appears to be common among quill mites. An exceptionally fascinating aspect of the evolutionary trajectory of syringophilids is the observation that the rate of speciation exhibits a remarkable degree of variability among the different genera within this group. For instance, when examining the Carduelini clade, one can observe that the species known as *Syringophilopsis kirgizorum* has a widespread distribution and is found inhabiting multiple species of hosts, in stark contrast to the genus *Syringophiloidus*, which is characterised by the presence of two species, *S. serini* and *S. klimovi*, that are both located within the same lineage of hosts. Such patterns of distribution and speciation suggest that the process of diversification among quill mites does not necessarily unfold in a manner that is synchronous with the evolutionary progress of their avian hosts. Instead, these apparent discrepancies may serve to illustrate the existence of intricate coevolutionary dynamics, wherein the speciation of parasites is influenced by a range of factors that extend beyond the mere phylogenetic relationships of their hosts.

Among other coevolutionary processes, we found no evidence of host switching; that is, no quill mite species in our dataset parasitising hosts outside the Fringillidae or coming from non-fringillid hosts. We also did not observe any cases of duplication or synhospitality cases (i.e., parasite speciation within a single host lineage [80]). However, we did identify likely sorting events, where parasite lineages have disappeared from certain host lineages, during the early stages of the diversification of the quill mite fauna within the Fringillidae (see Chapter 4.2). All these findings highlight the potential of quill mites as a model system for exploring host–parasite coevolutionary processes, even though much remains to be uncovered.

## 5. Conclusions

This research provides the most thorough evaluation to date of the syringophilinae biodiversity and host specificity within the avian family Fringillidae. We identified 20 distinct mite species, including 4 new to science, across 5 genera, parasitising 51 finch species belonging to all 3 subfamilies: Carduelinae, Euphoninae, and Fringillinae. The greatest diversity of quill mites was observed in the subfamily Carduelinae, which demonstrates the highest taxonomic richness concerning host species and genera. This association strongly suggests that the taxonomic diversity of hosts has a significant influence on the structure and complexity of parasite assemblages. Moreover, our results reveal that the majority of syringophiline mites associated with finches do not exhibit narrow host specificity. While five species (25%) are monoxenous, the majority are oligoxenous or mesostenoxenous, confined to hosts from phylogenetically related genera. In several instances, the presence of identical mite species among closely related hosts may indicate a delayed divergence of parasites relative to their hosts. Furthermore, significant differences in diversification rates among mite genera were noted; for example, Syringophilopsis displays extensive host ranges, while others, such as Syringophiloidus, seem to be more constrained. Such patterns of distribution and speciation suggest that the process of diversification among quill mites does not necessarily unfold in a manner that is synchronous with the speciation of their hosts.

Despite these advancements, substantial gaps in our understanding persist. A considerable percentage of Carduelinae genera, especially those that are monotypic or have few species, remains unsurveyed for quill mites. These unexamined lineages are likely to contain numerous undescribed species, and their inclusion in forthcoming studies will be crucial for refining estimates of parasite diversity and host specificity. Equally important is the expansion of research to encompass related avian families. Specifically, the Emberizidae, the sister group to the Fringillidae, remains notably understudied regarding quill mite associations, with only four host species analysed to date. This lack of data currently hinders meaningful comparisons of genus-level parasite assemblages between the two families or evaluations of shared evolutionary histories.

In summary, the Fringillidae–Syringophilinae system presents a compelling model for examining host–parasite co-phylogenetic patterns, host specificity, and parasite diversification over an extensive evolutionary timescale. However, achieving a more comprehensive understanding of these dynamics will necessitate enhanced sampling across underrepresented host taxa and a greater integration of morphological, ecological, and molecular data in future investigations.

## Figures and Tables

**Table 1:** Quill mites of the family Syringophilidae associated with birds of the family Fringillidae. AFRO—Afrotropic, PALA—Palaearctic, NEAR—Nearctic, SIJA—Sino-Japanese, NEOT—Neotropic, PANA—Panamanian, SAAR—Saharo-Arabian; *—type host species, c.p.—current paper. The ditto mark (″) indicates repetition of the species name.

Quill Mite Species	Host Species	Host Subfamily	Locality	References
***Aulobia* Kethley, 1970**				
*Aulobia cardueli* Skoracki, Hendricks and Spicer, 2010	*Acanthis flammea* (Linnaeus), Redpoll	Carduelinae	PALA: Poland, Slovakia, Germany	[42,51]
″	*Carduelis carduelis* (Linnaeus), European Goldfinch *	Carduelinae	PALA: Kazakhstan, Poland	[42,52]
″	*Carduelis citrinella* (Pallas), Citril Finch	Carduelinae	PALA: Poland	[42]
″	*Linaria flavirostris* (Linnaeus), Twite	Carduelinae	PALA: Poland, Germany	[42,51]
″	*Spinus psaltria* (Say), Lesser Goldfinch *	Carduelinae	NEAR: USA	[53]
″	*Spinus spinus* (Linnaeus), Eurasian Siskin	Carduelinae	PALA: Russia, Kazakhstan	[42]
*Aulobia leucostictus* Skoracki, 2011	*Leucosticte arctoa* (Pallas), Asian Rosy-Finch	Carduelinae	SIJA: Japan	([42], c.p.)
*Aulobia ruppelae* sp. n.	*Carpodacus roseus* (Pallas) Pallas’s Rosefinch *	Carduelinae	PALA: Russia	(c.p.)
″	*Carpodacus pulcherrimus* (Moore), Beautiful Rosefinch	Carduelinae	PALA: Nepal	(c.p.)
***Aulonastus* Kethley, 1970**				
*Aulonastus fringillus* Skoracki, 2011	*Fringilla coelebs* Linnaeus, Common Chaffinch	Fringillinae	PALA: Poland	[42]
*Aulonastus loxius* Skoracki, 2011	*Loxia curvirostra* Linnaeus, Red Crossbill *	Carduelinae	PALA: Poland	[42]
″	*Linaria cannabina* (Linnaeus), Eurasian Linnet	Carduelinae	PALA: Germany	([51], c.p.)
*Aulonastus neotropicalis* Sikora, Unsoeld, Melzer, Friedrich, Hromada and Skoracki, 2025	*Chlorophonia cyanea* (Thunberg), Blue-naped Chlorophonia *	Euphoninae	NEOT: Venezuela, Bolivia	[55]
″	*Chlorophonia cyanocephala* (Vieillot), Golden-rumped Euphonia	Euphoninae	NEOT: Colombia	[55]
″	*Chlorophonia flavifrons* (Sparrman), Lesser Antillean Euphonia	Euphoninae	PANA: Guadeloupe (Lesser Antillean Creole)	[55]
″	*Euphonia affinis* (Lesson), Scrub Euphonia	Euphoninae	NEOT: Colombia	[55]
″	*Euphonia concinna* Sclater, Velvet-fronted Euphonia	Euphoninae	NEOT: Colombia	[55]
″	*Euphonia luteicapilla* (Cabanis), Yellow-crowned Euphonia	Euphoninae	PANA: Panama	[55]
″	*Euphonia anneae* Cassin, Tawny-capped Euphonia	Euphoninae	NEOT: Costa Rica	[55]
″	*Euphonia xanthogaster* Sundevall, Orange-bellied Euphonia	Euphoninae	NEOT: Peru	[55]
″	*Euphonia chrysopasta* Sclater and Salvin, White-lored Euphonia	Euphoninae	NEOT: Venezuela	[55]
″	*Euphonia cayennensis* (Gmelin), Golden-sided Euphonia	Euphoninae	NEOT: French Guiana	[55]
*Aulonastus ritzerfeldi* sp. n.	*Leucosticte arctoa* (Pallas), Asian Rosy-Finch	Carduelinae	SIJA: Japan	(c.p.)
***Syringophiloidus* Kethley, 1970**				
*Syringophiloidus coccothraustes* Skoracki, 2011	*Coccothraustes coccothraustes* (Linnaeus), Hawfinch	Carduelinae	PALA: Poland	[42]
*Syringophiloidus carpodaci* Bochkov and Apanaskevich, 2001	*Carpodacus erythrinus* (Pallas) Common Rosefinch *	Carduelinae	PALA: Poland, Kazakhstan, Russia, Nepal	([42,56], c.p.)
″	*Carpodacus sipahi* (Hodgson), Scarlet Finch	Carduelinae	PALA: Nepal	(c.p.)
*Syringophiloidus klimovi* Skoracki and Bochkov, 2010	*Chloris chloris* (Linnaeus), European Greenfinch *	Carduelinae	PALA: Kazakhstan, England	[42,52]
″	*Rhodospiza obsoleta* (Lichtenstein), Desert Finch	Carduelinae	PALA: Germany	[51]
*Syringophiloidus serini* Bochkov, Fain and Skoracki, 2004	*Crithagra atrogularis* (Smith), Yellow-rumped Seedeater	Carduelinae	AFRO: Tanzania	(c.p.)
″	*Crithagra burtoni* (Gray), Thick-billed Seedeater	Carduelinae	AFRO: Tanzania	(c.p.)
″	*Crithagra citrinelloides* (Rüppell), African Citril	Carduelinae	AFRO: Tanzania	(c.p.)
″	*Crithagra mozambica* Müller, Yellow-fronted Canary *	Carduelinae	AFRO: Central Africa	[57]
″	*Linaria flavirostris* (Linnaeus), Twite	Carduelinae	PALA: Kyrgyzstan	(c.p.)
″	*Serinus flavivertex* (Blanford), Yellow-crowned Canary	Carduelinae	AFRO: Tanzania	(c.p.)
*Syringophiloidus stawarczyki* Skoracki, 2004	*Chlorophonia cyanocephala* (Vieillot), Golden-rumped Euphonia *	Euphoninae	NEOT: Brazil, Colombia, Paraguay	[55,58]
″	*Chlorophonia cyanea* (Thunberg), Blue-naped Chlorophonia	Euphoninae	NEOT: Brazil, Colombia	[55]
″	*Euphonia chlorotica* (Linnaeus), Purple-throated Euphonia	Euphoninae	NEOT: Paraguay	[55]
″	*Euphonia chrysopasta* (Sclater and Salvin), White-lored Euphonia	Euphoninae	NEOT: Venezuela, Bolivia	[55]
″	*Euphonia concinna* (Sclater), Velvet-fronted Euphonia	Euphoninae	NEOT: Colombia	[55]
″	*Euphonia laniirostris* (d’Orbigny and Lafresnaye), Thick-billed Euphonia	Euphoninae	NEOT: Venezuela	[55]
″	*Euphonia violacea* (Linnaeus), Violaceous Euphonia	Euphoninae	NEOT: Trinidad and Tobago	[55]
***Syringophilopsis* Kethley, 1970**				
*Syringophilopsis euphonicus* Sikora, Unsoeld, Melzer, Friedrich, Hromada and Skoracki, 2025	*Euphonia trinitatis* Strickland, Trinidad Euphonia *	Euphoninae	NEOT: Venezuela	[55]
″	*Euphonia xanthogaster* Sundevall, Orange-bellied Euphonia	Euphoninae	NEOT: Peru	[55]
″	*Euphonia minuta* Cabanis, White-vented Euphonia	Euphoninae	NEOT: Colombia	[55]
*Syringophilopsis fringillae* (Fritsch, 1958)	*Fringilla coelebs* Linnaeus, Common Chaffinch *	Fringillinae	PALA: England, Germany, Poland, Slovakia, Russia, Kazakhstan	[42,52,58,59,60,61,62]
″	*Fringilla montifringilla* Linnaeus, Brambling	Fringillinae	PALA: Germany	[51]
*Syringophilopsis kirgizorum* Bochkov, Mironov and Kravtsova, 2000	*Carduelis carduelis* (Linnaeus), European Goldfinch	Carduelinae	PALA: Poland, Russia	[42,58]
″	*Chloris chloris* (Linnaeus), European Greenfinch *	Carduelinae	PALA: Kyrgyzstan, Poland, England, Germany SAAR: Jordan	[42,51,58,63,64,65]
″	*Linaria cannabina* (Linnaeus), Eurasian Linnet	Carduelinae	SAAR: Jordan	[65]
″	*Linurgus olivaceus* (Fraser), Oriole Finch	Carduelinae	AFRO: Cameroon	[66]
″	*Rhodospiza obsoleta* (Lichtenstein), Desert Finch	Carduelinae	PALA: Kyrgyzstan	[63]
***Torotrogla* Kethley, 1970**				
*Torotrogla cardueli* Bochkov and Mironov, 1999	*Carduelis carduelis* Linnaeus, European Goldfinch	Carduelinae	PALA: Poland	[42]
″	*Linaria cannabina* (Linnaeus), Common Linnet	Carduelinae	PALA: Poland, Slovakia	[42]
″	*Loxia curvirostra* Linnaeus, Red Crossbill	Carduelinae	PALA: Poland	[42]
″	*Loxia leucoptera* Gmelin, White-winged Crossbill	Carduelinae	PALA: Poland	[42]
″	*Loxia pytyopsittacus* Borkhausen, Parrot Crossbill	Carduelinae	PALA: Finland	[42]
″	*Serinus canaria* (Linnaeus), Atlantic Canary	Carduelinae	PALA: Europe	[42]
″	*Serinus pusillus* (Pallas), Fire-fronted Serin	Carduelinae	PALA: Kyrgyzstan	(c.p.)
″	*Spinus spinus* (Linnaeus), Eurasian Siskin *	Carduelinae	PALA: Poland, Russia, Slovakia	[42,67]
*Torotrogla coccothraustes* Bochkov, Flannery and Spicer, 2009	*Coccothraustes vespertinus* (Cooper), Evening Grosbeak	Carduelinae	NEAR: USA	[68]
*Torotrogla gaudi* Bochkov and Mironov, 1998	*Fringilla coelebs* Linnaeus, Common Chaffinch *	Fringillinae	PALA: Poland, Russia	[42,60]
″	*Fringilla montifringilla* Linnaeus, Brambling	Fringillinae	PALA: Poland, Slovakia	[42,69]
*Torotrogla enucleator* sp. n.	*Pinicola enucleator* (Linnaeus), Pine Grosbeak *	Carduelinae	SIJA: Japan	(c.p.)
″	*Leucosticte nemoricola* (Hodgson), Plain Mountain-Finch	Carduelinae	PALA: Kyrgyzstan	(c.p.)
*Torotrogla janhafti* sp. n.	*Carpodacus rhodochlamys* (Brandt), Red-mantled Rosefinch *	Carduelinae	PALA: Kyrgyzstan	(c.p.)
″	*Carpodacus sibiricus* (Pallas), Siberian Long-tailed Rosefinch	Carduelinae	SIJA: Japan	(c.p.)
″	*Carpodacus synoicus* (Temminck), Sinai Rosefinch	Carduelinae	SAAR: Egypt	(c.p.)
″	*Carpodacus thura* Bonaparte and Schlegel, Himalayan White-browed Rosefinch	Carduelinae	PALA: Nepal	(c.p.)

## Data Availability

All necessary data are available in the text.
